# Letter from the Editor in Chief

**DOI:** 10.19102/icrm.2020.111206

**Published:** 2020-12-15

**Authors:** Moussa Mansour


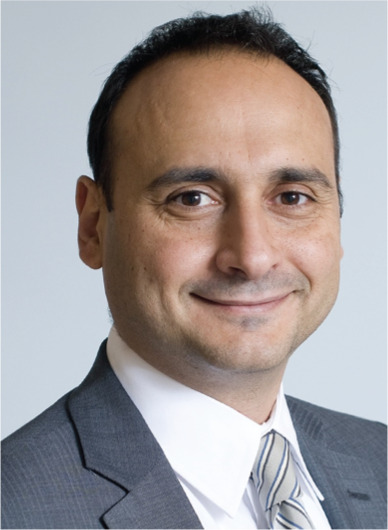


Dear Readers,

The coronavirus disease 2019 (COVID-19) pandemic has adversely affected the field of cardiac electrophysiology in a significant way. Most tragic has been the loss of colleagues, friends, and relatives. Many hospitals in heavily affected areas have halted some or all elective procedures. Finally, scientific conferences were either canceled or changed to a virtual format and in-person patient visits have been modified to the same.

However, the COVID-19 pandemic has also accelerated the adoption of some beneficial trends, one of which is same-day discharge after electrophysiology procedures, which has served to free up hospital beds and reduce the risk of viral infection. Early mobilization and same-day discharge after cardiac implantable electronic device placement have been practiced in some centers for many years, albeit excluding high-risk patients such those at risk for bleeding or on oral anticoagulation, those with complete heart block, those with secondary-prevention implantable cardioverter-defibrillator indications, and those experiencing immediate procedural complications. Studies have shown that this approach may lead to greater patient satisfaction and cost savings without adversely affecting readmission rates.^1,2^ Also, recent advances in technology have allowed the safe discharge of patients after atrial fibrillation ablation; these include the use of vascular ultrasound to guide vascular access, intracardiac echocardiography to reduce the risk of cardiac perforation and assess the pericardial space at the end of the procedure, uninterrupted oral anticoagulation, improved ablation technologies allowing for faster pulmonary vein isolation, and venous closure devices to facilitate patient mobilization just two hours after the procedure. There is significant evidence supporting this practice and research suggests that same-day discharge after atrial fibrillation ablation is safe and not associated with higher hospital readmission or complication rates after discharge.^3^

As the COVID-19 pandemic continues, pursuing same-day discharge where appropriate may help us to limit the effects of this public health crisis on the field of cardiac electrophysiology and continue to provide our patients with safe and timely care.

Best wishes for a happy holiday season.

Sincerely,


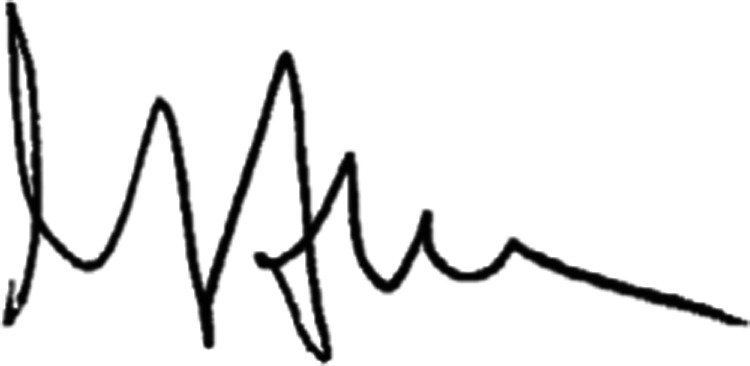


Moussa Mansour, MD, FHRS, FACC

Editor in Chief

The Journal of Innovations in Cardiac Rhythm Management

MMansour@InnovationsInCRM.com

Director, Atrial Fibrillation Program

Jeremy Ruskin and Dan Starks Endowed Chair in Cardiology

Massachusetts General Hospital

Boston, MA 02114
